# Fermentation of cocoa pod husks with *Pleurotus salmoneo‐stramineus* for food applications

**DOI:** 10.1002/fsn3.3937

**Published:** 2024-01-12

**Authors:** Thomas Bickel Haase, Victoria Klis, Andreas Klaus Hammer, Claudia Pinto Lopez, Christoph Verheyen, Susanne Naumann‐Gola, Holger Zorn

**Affiliations:** ^1^ Fraunhofer Institute for Process Engineering and Packaging IVV Freising Germany; ^2^ Institute of Food Chemistry and Food Biotechnology, Justus‐Liebig University Giessen Germany; ^3^ Fraunhofer Institute for Molecular Biology and Applied Ecology IME Giessen Germany

**Keywords:** cocoa fruit, ergosterol, fermented by‐products, fungal mycelium, techno‐functional properties

## Abstract

Cocoa pod husks (CPHs), the major side‐stream from cocoa production, were valorized through fermentation with *Pleurotus salmoneo‐stramineus* (PSS). Considering ergosterol as a biomarker for the fungal content, the mycelium accounted for 54% of the total biomass after 8 days in submerged cultures. The crude protein content of fermented CPH (CPHF) increased from 7.3 g/100 g DM in CPH to 18.9 g/100 g DM. CPH fermentation resulted in a high biological value of 86 for the protein. The water and oil binding capacities of CPHF were 3.5 mL/g and 2.1 mL/g, respectively. The particle diameter d_v,0,90_ of CPHF was 373 μm as compared to 526 μm for CPH. The total dietary fiber was 73.4 g/100 g DM in CPHF and 63.6 g/100 g DM in CPH. The amount of soluble fiber was 2.3 g/100 g DM in CPHF and 10.1 g/100 g DM in CPH; the insoluble fraction accounted for 71.1 g/100 g DM and 53.6 g/100 g DM, respectively. Bread doughs with CPH or CPHF were characterized for texture, color, and farinographic properties. The dough hardness, consistency, and browning index increased with the concentration of CPH, whereas for CPHF, springiness and peak viscosities declined. We demonstrate the upcycling of CPH into nutritious and functional ingredients through PSS fermentation.

## Graphical abstract

The manuscript reports on the upcycling of cocoa pod husks by fermentation with the basidiomycetous fungus *Pleurotus salmoneo‐stramineus*. The fermentation resulted in a high biological value of 86 for the protein and promising techno‐functional properties in farinographic and baking experiments.

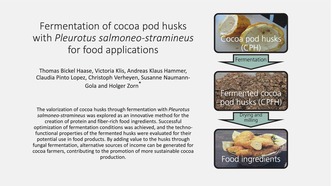

## 1 INTRODUCTION

With the introduction of the 17 Sustainable Development Goals, the UN highlighted the importance of facing poverty and other deprivations with strategies that promote health, education, equality, and strengthen economic growth. Accordingly, there is a growing demand for strategies to create safe and nutritious foods (United Nations, 2022). Therefore, effort should be made on the transformation of agricultural side streams, such as those from the cocoa industry, into nutritious and functional foods. In the present study, we propose the fungal fermentation of cocoa pod husks as an innovative approach to creating fiber‐ and protein‐rich food ingredients.

The production of cocoa beans is forecasted for 2022/2023 to amount to approximately 5.0 million tons (International Cocoa Organization, 2022). The cocoa beans contribute to approximately 10% of the total mass of the fruit, while the cocoa pod husk (CPH) represents the major fraction with about 70%—80% (Prabhakaran Nair, 2010). The large amounts of husks pose severe disposal challenges, as the uncontrolled accumulation may lead to the transmission of cocoa pests and diseases (Guest, 2007). CPH comprises the epicarp, mesocarp, endocarp, and a sclerotic portion (Campos‐Vega et al., 2018). CPH has been reported to contain 76.6 g/kg crude protein in the dry matter, 325 g/kg crude fiber, and 101 g/kg ash. The energy content is 4.72 MJ/kg. CPHs consist mainly of fibrous materials, including 19.7%—26.1% cellulose, 8.7%—12.8% hemicelluloses, 14%—28% lignin, and 6.0%—12.6% pectin (Donkoh et al., 1991). The high proportion of lignin in CPH results in a woody, coarse texture. This makes the husks hardly digestible for humans and impedes their direct use in food products. Nonetheless, CPH contains soluble and insoluble dietary fiber, which could be interesting for the food sector. Some other applications for the use of CPH have been described. For instance, CPHs have been used in animal feed (Donkoh et al., 1991; Sobamiwa & Longe, 1994), as a feedstock for soap making (Gyedu‐Akoto et al., 2015), and as an alternative energy source (Agyeman & Oldham, 1986). Biotechnological uses of CPH include its incorporation into food systems (Vriesmann et al., 2011) and biofuel production (Vásquez et al., 2019). Nevertheless, suitable transformation technologies are necessary to make cocoa pods accessible for human nutrition and food applications.

In recent years, the fermentation of side streams by higher mushrooms of the Basidiomycota division has gained increasing attention. The Basidiomycota include about 30,000 described species, which comprise, among others, fruiting body‐forming fungi (Watkinson et al., 2016). The fruiting bodies of several edible mushrooms have become popular foods mainly due to their pleasant taste and their beneficial nutritional properties. Some of the health‐promoting characteristics ascribed to them are the high‐quality protein, the high fiber content, the presence of vitamin D_2_ and several B vitamins, as well as their low fat content (Ahlborn et al., 2018; Manzi et al., 1999). Their high protein content makes them especially interesting as vegan protein sources (Stephan et al., 2018). As an alternative to the traditional production of fruiting bodies, fungal mycelia can be cultivated in submerged fermentations in the form of mycelial pellets (Ahlborn et al., 2019; Trapp et al., 2018). The mushroom mycelium exhibits similar positive nutritional properties as the fruiting bodies and is considered a promising alternative to plant‐based proteins (Stephan et al., 2018). High biological values have been reported for fungal mycelial proteins previously (Ahlborn et al., 2019). In addition, mycelium growth has been shown to depend, among other things, on the carbon to nitrogen ratio, the type of carbon or nitrogen source, and the temperature (Hoa et al., 2015; Hoa & Wang, 2015). The application of mycelium from side stream fermentations can be performed in different ways: the processing of the mycelium–substrate complex as a whole, the mycelium separated from the substrate, the protein extracted from the mycelium, and the protein secreted by the fungus (Scholtmeijer et al., 2023). Furthermore, the fermentation of higher mushrooms offers possibilities to improve the organoleptic properties of agricultural side‐streams, such as by‐products from the cocoa industry (Klis et al., 2023).

Fermentation of CPH by fungi might be an interesting approach to creating protein‐ and fiber‐rich ingredients for the food industry, thereby increasing the sustainability of the cocoa supply chain and addressing the challenges caused by the constantly growing world population. By giving additional value to CPH, cocoa farmers may benefit from new sources of income and better livelihoods, fomenting a more sustainable cocoa production (Vásquez et al., 2019) and thereby complying with the Sustainable Development Goals. This study explores possibilities for the valorization of CPH by fermentation with *Pleurotus salmoneo‐stramineus* (PSS). The fermentation conditions were optimized, and the techno‐functional properties of the fermented cocoa pod husks (CPHF) were evaluated for their use in food products using enriched bread doughs as demonstrators.

## 2 MATERIALS AND METHODS

### 2.1 Fermentation of cocoa pod husks with *Pleurotus salmoneo‐stramineus*

The fermentation was performed according to Bosse et al. (2013) and Trapp et al. (2018). CPH from the cocoa variety SUL1 were obtained in 2020 from the cocoa germplasm collection garden of the Indonesian Coffee and Cocoa Research Institute (ICCRI), located in Nogosari village, Jember Regency, East Java. Prior to processing, the cocoa pod husks were washed thoroughly with water and left to dry at room temperature. A fine‐grained CPH powder was obtained by drying, crushing, and homogenizing CPH. Using a Retsch SM 2000 (Retsch GmbH), the sun‐dried husks were milled and sieved to a particle diameter of less than 1—2 mm. The ground CPH was stored at room temperature. Prior to fermentation, CPH were autoclaved to inhibit the growth of undesirable microorganisms. PSS was obtained from the Institute for Molecular Wood Biotechnology and Technical Mycology, Göttingen, Germany, and was selected from a total of 43 screened basidiomycetes as the most promising fungus for further analysis. For strain maintenance, the fungus was kept on malt extract agar plates (20 g/L malt extract, 15 g/L agar agar) and transferred to a new plate every 6 days using a spatula and by cutting out a 1 cm^2^ piece of overgrown agar. For the pre‐cultures, 200 mL of malt extract medium (20 g/L malt extract in drinking water) was placed in a 500 mL narrow‐neck Erlenmeyer flask and inoculated with 1 cm^2^ of mycelium. Homogenization was performed by using an Ultraturrax (IKA Works Inc.) at 10,000 rpm for 30 s. Cultivation took place on a horizontal shaker at 150 rpm at 24°C in the dark for 6 days. To ensure the comparability of the pre‐cultures, these conditions were replicated between the different approaches. The main cultures were based on CPH medium consisting of 20 g/L finely ground husks and 3.6 g/L mono‐sodium aspartate as a nitrogen supplement. 2 L of the main culture medium was added to a 5 L Erlenmeyer flask. The pre‐culture was homogenized using an Ultraturrax, and 200 mL was added to the main culture flask. A fermentation time of 8 days was selected. Finally, the harvested material was passed through a cheese cloth to separate the mycelium–CPH composite (CPHF) from the supernatant. This was washed with drinking water, and the resulting biomass was dried by lyophilization.

### 2.2 Estimation of the fungal content via quantitation of the biomarker ergosterol

Freeze‐dried mycelia were ground for 3 min in a vibrating mill (25 Hz) (MM 400, Retsch GmbH), and approximately 50 mg was weighed into a pyrex™ tube. After addition of 25 mg sodium ascorbate as an antioxidant, 0.25 mL internal standard (IST) (0.5 mg/mL 7‐dehydrocholesterol ((>95% Sigma‐Aldrich Chemie GmbH, Taufkirchen, Germany) in 2‐butanone), and 5 mL methanolic NaOH (5% NaOH in 95% methanol)), the samples were vortexed and saponified for 60 min at 80°C in a water bath. After cooling in the dark, the samples were filtered through a membrane filter (0.45 μm (LLG‐Labware, Meckenheim, Germany)) and extracted three times with 5 mL of n‐hexane. The organic phases were combined in a 15 mL volumetric flask, made up *ad* mark with n‐hexane, and dried over Na_2_SO_4_. Six milliliter of the sample (dilution factor = 2.5) was transferred to a fresh pyrex™ tube and dried under an N_2_‐stream. The residue was dissolved in 0.5 mL of tetrahydrofuran (THF) and 0.5 mL of N‐methyl‐N‐(trimethylsilyl) trifluoroacetamide (MSTFA) (Fluorochem Ltd.) and vortexed. After incubation at 70°C for 2 min, silylation was performed at room temperature overnight. Quantitation was performed by external calibration via the IST in the range of 10–100 μg/mL. Therefore, an ergosterol stock solution of 200 μg/mL (>95% TCI Deutschland GmbH) was prepared in 2‐butanone. The individual calibration standards were prepared in 10 mL volumetric flasks by adding 1 mL of IST and between 0.5 and 5.0 mL of ergosterol stock solution *ad* 10 mL. One mL of the samples was concentrated to dryness under N_2_ and then treated in the same way as the samples. For quantitative estimation of the fungal content, the ergosterol value of CPHF was compared to the reference value for pure PSS mycelium grown in malt extract medium (434 ± 31 mg/100 g), which was set to 100%.

Gas chromatographic (GC‐FID) analysis was performed using a gas chromatograph (GC) (Agilent 7890, Agilent Technologies Inc) equipped with an auto sampler (Agilent 7683B) and a flame ionization detector (FID). One microliter of the sample was injected at 250°C via a split/splitless injector in splitless mode. A 30 m × 0.32 mm × 0.25 μm DB5ms column (Agilent Technologies, 123–5532) was used. The oven temperature program started at 100°C for 3 min, was heated to 280°C with 30°C/min, held for 12 min, heated to 320°C with 30°C and held for another 5 min. H_2_ was used as carrier gas at a constant flow rate of 2.2 mL/min. The flow rates of gases in the FID were set as follows: H_2_: 40 mL/min; air: 400 mL/min; and makeup gas (N_2_): 25 mL/min. The method was validated regarding linearity, recovery, method precision, limit of detection (LOD), and limit of quantification (LOQ) following DIN 32645.

### 2.3 Characterization of CPH and CPHF

Prior to the chemical characterization, the wet CPHF mass was freeze‐dried and milled to obtain a homogeneous sample. Milling was carried out with a Grindomix GM 200 (Retsch GmbH) for 15 s at 7500 rpm. The non‐fermented, dried, and milled CPH was used for comparison. The composition of wheat flour type 550 from Mühlen König (Frießinger Mühle GmbH) was also analyzed as it was used in the development of bread doughs.

#### 2.3.1 Determination of the dry matter content

Samples were dried in a LECO TGA 601 (LECO Instrumente GmbH) oven in a ceramic crucible at 105°C (Lebensmittel‐ und Futtermittelgesetzbuch, 2021). The dry matter (DM) was determined gravimetrically by differential weighing.

The residual moisture of fermentates during optimization of the growth time of PSS on CPH was determined with a moisture analyzer (MA 35, Sartorius AG).

#### 2.3.2 Determination of the crude protein and ash contents

The crude protein content of the samples was derived from the nitrogen content determined according to the Dumas method. This method can be applied to all foodstuffs and animal feed in a range of 1.9—23.9 mg N absolute in solid samples and is described in § 64 LFBG (2013). Analyses were carried out with a TruMac N Nitrogen Determinator (LECO Instrumente GmbH). The crude protein contents of the PSS fermentates during optimization trials were determined according to Kjeldahl as described in AOAC 2001.11 (AOAC, 2005). In both cases, to calculate the protein content of the samples, the nitrogen content was multiplied by the protein factor 6.39 for CPH and 6.00 for CPHF obtained from the amino acid distribution (2.3.3).

The ash was determined by means of LECO TGA 601 (LECO Instrumente GmbH). The samples were incinerated in a ceramic pan at 550°C (Lebensmittel‐ und Futtermittelgesetzbuch, 2021). The ash content was determined gravimetrically by differential weighing.

#### 2.3.3 Determination of amino acids and calculation of the biological value

Amino acid analyses were carried out, according to Ahlborn et al. (2019). Approx. 30 mg of CPH or CPHF was mixed with 2.5 mL of 6 M HCl (containing 1 g/L phenol) and incubated at 110°C for 24 h for total hydrolysis. After cooling, 1.5 mL of sodium hydroxide solution (7.5 M) was added, and the pH was carefully adjusted to 2.2 with 7.5 and 1 M sodium hydroxide solutions. The volume was adjusted to 20 mL with citrate buffer (11 g trisodium citrate dihydrate, 6 g citric acid, 14 mL thiodiglycol, 12 mL of a 32% (*w/w*) HCl, and 2 g phenol made up to 1 L with *dd*H_2_O and adjusted to pH 2.2) in a volumetric flask, and the solution was filtered through a syringe filter (0.45 μm) into a vial. For determination of cysteine and methionine, oxidation was performed prior to the hydrolysis with 0.5 mL oxidation mix (0.05 mL hydrogen peroxide (w = 30%), mixed with a 0.45 mL phenolic formic acid (889 g formic acid mixed with 111 g *dd*H_2_O, containing 4.73 g phenol)) for 16 h at 4°C. The oxidation was stopped by the addition of 0.084 g of sodium disulfite. For quantitation of tryptophane, an alkaline hydrolysis was carried out with 2.5 mL of phenolic sodium hydroxide solution (5 M sodium hydroxide solution containing 0.1% phenol). After the addition of 1.5 mL of 0.5 M phosphoric acid, the pH was adjusted to 2.2 with 3.75 M and 1 M HCl.

Amino acid analysis was carried out with an amino acid analyzer S433 (Sykam GmbH) with an LCA K13/Na and a gradient program of two sodium citrate buffer solutions (A: 0.12 N, pH 3.45; B: 0.20 N, pH 10.85) and a regeneration solution (20 g sodium hydroxide and 0.2 g EDTA per liter *dd*H_2_O) with a flow rate of 0.45 mL/min. Post‐column derivatization was carried out with 0.2 M ninhydrin (pH 10.85) with a flow of 0.25 mL/min. The injection volume was 150 μL. Identification and quantification were carried out by external calibration in a range of 10—200 nmol/mL with an amino acid calibration mix (Sykam GmbH, Fürstenfeldbruck, Germany) (R^2^_all amino acids_ >0.999). The calibration mix (standard solution H‐Ox, Sykam) contained all amino acids, including Cys‐Ox and Met‐Ox, except for tryptophane. A tryptophane stock solution (L‐tryptophane ≥99% Carl Roth GmbH + Co. KG) was prepared separately.

To calculate the pure protein content, the AA_res_ value was calculated for each amino acid, taking into account the loss of one water molecule per peptide bond. The sum of all AA_res_ gives the pure protein content. To evaluate the protein quality, the biological value (BV) was calculated via the essential amino acid index (EAAI) (Ahlborn et al., 2019), related to the reference protein defined by FAO/WHO (1973) with a BV of 100.

Based on the determined amino acid profiles, corrected nitrogen to protein conversion factors were calculated for CPH (F_CPH_ = 6.39) and CPHF (F_CPHF_ = 6.00).

#### 2.3.4 Determination of total dietary fibers

The determination of the total dietary fiber content as well as the contents of soluble and insoluble dietary fiber was carried out according to the AOAC 991.43 (Association of Analytical Chemists [AOAC], 2000) method using an enzyme assay kit (K‐TDFR‐200A, Megazyme Ltd).

#### 2.3.5 Scanning electron microscopy

Scanning electron microscopy was performed under high‐vacuum using an EVO LS 10 SEM (Zeiss, Jena, Germany), equipped with a secondary electron detector (Everhart–Thornley detector). The excitation voltage was 15.00 kV. All samples were sputtered with gold.

#### 2.3.6 Determination of the water‐binding capacity

The water‐binding capacity was determined by combining 2.0 g of sample with an approximately 20‐fold excess of water. After mixing well and 24 h of soaking, the samples were centrifuged at 2500 *g*, at 20°C for 5 min. After centrifugation, the supernatant (unbound water) was discarded, and the samples were turned upside down and drained for 25 min. The weight of the water‐saturated sample was determined, and the water‐binding capacity [g/g] was calculated (American Association of Cereal Chemists [AACC], 1978).

#### 2.3.7 Determination of the oil binding capacity

The oil binding capacity (OBC) of CPH, CPHF, and flour was analyzed using the method described by Ludwig et al. (1989) at room temperature (~21°C). In scaled centrifuge tubes, a 1.5 g sample was dispersed in an excess of corn oil (10 mL). After vigorously mixing, the sample was centrifuged at 700 *g* and 20°C for 15 min. The volume of free oil was read on the side of the centrifuge tube and calculated as described by Ludwig et al. (1989).

#### 2.3.8 Particle size distribution

The particle size distributions of flour, CPH, and CPHF were measured using a static laser diffraction instrument (Malvern Mastersizer 3000, Software version 2.15, Malvern Instruments Ltd). The refractive index of the particles was determined to be 1.53, the absorbance was 0.005 [L/mol cm], and the refractive index of the dispersant was 1.40. The particles were irregularly shaped, and the Mie theory was applied as an optical model. The samples were mixed with butanol in a 1:1 ratio, equilibrated to room temperature, and then added to the dispersing unit. Butanol (99.4%, Sigma Aldrich, Merck KGaA) was used as the dispersant. To standardize the sample concentration, the obscuration was adjusted between 10% and 15%. The stirring speed of the dispersion unit was set to 3000 min^−1^. To ensure a homogenous dispersion of the sample, measurements were started after 2 min and repeated 1 min later. Samples were measured in triplicate. As the stirring did not affect the particle sizes (second measurement), all six values were taken into consideration when calculating the means. The particle size measurements are reported as d_v,0.1_, d_v,0.5,_ and d_v,0.9_. The diameters d_v,0.1_, d_v,0.5,_ and d_v,0.9_ correspond to 10, 50, and 90 vol% on a relative cumulative particle size curve, respectively.

#### 2.3.9 Determination of protein solubility

1.50 g of sample was added to 35 mL of a 0.1 mol/L sodium chloride solution while stirring until it was suspended. The initial pH value was noted and then adjusted to 7.0. The samples were held at this pH value for 1 h and the pH value was measured after 30 min and 1 h. The suspension was then transferred to a 50 mL volumetric flask and filled up to the mark. After vigorous shaking, 20 mL of the solution was centrifuged for 15 min at 20,000 *g* and 15°C. The supernatant was filtered through a Whatman No.1 filter (Whatman GmbH), and the nitrogen content was determined in the filtrate after Dumas (1831) (2.2.3). The protein content was calculated with the protein factor 6.39 for CPH, 6.00 for CPHF, and 5.81 for wheat flour. The protein solubility was determined using Equation 1:

(1) $$\begin{array}{c} \text{Protein solubility}\;\left[\%\right]=\\ \;\frac{\text{Volume NaCl solution}\;\left[\mathrm{mL}\right]*\text{Protein in the supernatant}\;\left[\frac{\mathrm{mg}}{\mathrm{mL}}\right]\;}{\text{Sample mass}\;\left[\mathrm{mg}\right]*\text{Protein in the}\;DM\;\left[\%\right]\;x\;\mathrm{DM}\;\text{of the sample}\;\left[\%\right]}*100 \end{array}$$

#### 2.3.10 Determination of color and browning index

The DigiEye color imaging system (DigiEye V2.62, VeriVide), comprising an illumination box with diffuse illuminant D65 and a Nikon D90 digital camera, was used for color determinations. Digitizer calibration charts were used to calibrate the system. For the color measurements, the sample was evenly distributed in a white sample cup (Aqualab, Meter Group), and the average surface color was expressed as CIE L*a*b*‐values with L*, a*, and b* ranging from black (0) to white (100), from green (−128) to red (+127), and from blue (−128) to yellow (+127), respectively (Kumah et al., 2019). The browning index was calculated using the L*a*b* values and following the formula described in Bal et al. (2011).

#### 2.3.11 Determination of pasting properties

The AACC 76‐21 500 test was conducted to evaluate the viscosity of the samples in excess of water during temperature‐controlled cycles (pasting properties) (AACC, 2000). For this analysis, the RVA 4500 (PerkinElmer Inc) with paddle stirrers (PerkinElmer) was used.

All measurements were carried out in triplicate. Three different CPH and CPHF concentrations (2.5% (*w/w*), 5% (*w/w*) and 10% (*w/w*)) were added to the wheat flour. The pure flour as well as the CPH and CPHF were also investigated. A total of 3.5 g of flour‐blend was weighted into the sample cups and mixed with 25 mL of water until the sample was homogenously suspended. Next, the sample cup was assembled into the RVA, and the test was immediately started.

### 2.4 Analysis of white bread dough with added CPH and CPHF

Different dough samples were prepared by substituting proportional weight amounts (0% (*w/w*) (standard wheat bread dough), 2.5% (*w/w*), 5% (*w/w*), and 10% (*w/w*)) of flour Type 550 with CPH and CPHF. Doughs were prepared by adding 53.5% (*w/w*), water. No further ingredients, such as salt or yeast, were added to the blends to keep the dough as simple as possible and to enable the comparison between samples.

#### 2.4.1 Farinographic measurements

As wheat flours and their capacities for water uptake may differ between producers and production batches, the water absorption of the flour and the resistance of the various doughs to mixing were determined following the International Association for Cereal Science and Technology (ICC) Standard No. 115/1 (International Association for Cereal Science and Technology, 1992). With a farinograph (Successor Farinograph TS, Brabender GmbH & Co. KG), the kneading curve was plotted in a force–time diagram from which parameters such as the optimal water absorption, dough development time, dough stability, and dough softening were derived. To determine the optimal water absorption of the wheat flour type 550, a dough with a consistency of 500 Farinograph Units (FE) was prepared in a 50 g measuring cell using sigma blades. The value 500 FE was chosen, as it is a popular and desired value for the texture and dough performance of bread doughs (Miś et al., 2012). Measurements were conducted at 30°C for 20 min. The optimal water absorption of the wheat flour accounted for 53.5% (*w/w*). The amount of water added to the flour blends was kept constant to determine how the addition of CPH or CPHF affected the doughs' properties. The water content of the flour was determined to be 13.7%, while the water contents of the flour blends were calculated, taking the DM of CPH and CPHF as well as their concentrations in the blends into account. These values were entered into the equipment's software prior to measurements. The obtained doughs were evaluated based on their textural profiles (2.4.2).

#### 2.4.2 Texture profile analysis (TPA)

A texture analyzer (TA. XT plus Stable Micro Systems) was used to assess the hardness and springiness of the dough samples made with different weight percentages of CPHF and CPH. 20 g of dough was carefully rolled into a ball and left 10 min to rest in closed vessels to prevent drying out. The TPA analyses consisted of two cyclic compression steps of 40% deformation. A Plexiglas cylinder probe with a diameter of 25 mm (TA. XT plus Stable Micro Systems) was chosen. The hardness was defined as the peak force in Newton [N] during the first compression cycle, whereas the springiness described the height that the dough recovered during the time elapsed between compressions (Bourne, 1978).

### 2.5 Statistics

The results were expressed as the mean ± standard deviation (SD). Statistical analysis was performed using Tukey's multiple comparison test (*p* < .05) to determine significant differences between two groups using Origin 2022b (OriginLab Corporation). With the same software, Spearman's list‐wise correlation analysis was performed to establish associations between the results.

## 3 RESULTS AND DISCUSSION

### 3.1 Fermentation of CPH with *Pleurotus salmoneo‐stramineus*

#### 3.1.1 Validation of the GC‐FID method for quantitation of ergosterol

The method was validated regarding linearity in a range of 10—100 μg/mL, corresponding to 50—500 mg/100 g (*n* = 3; *R* ^2^ = .9999). The linearity test, according to Mandel, proved the linearity in the given range. The average recovery in the CPH matrix at ten points in the same range was between 97.016% and 101.83% (*n* = 3). The method precision showed a relative standard deviation (RSD) of 4.0%. The LOD determined by means of the calibration method following DIN 32645 was 0.42 ± 0.11 μg/mL respectively 2.1 mg/100 g (*n* = 3) and the LOQ was 1.44 ± 0.38 μg/mL respectively 7.2 mg/100 g (*n* = 3). Considering the ergosterol contents of various basidiomycetes (e.g. 210 mg/100 g DM for *Phanerochaete chrysosporium* or 375 mg/100 g DM for *Pleurotus sapidus*, depending on culture media and fermentation time) described in the literature (Ahlborn et al., 2018; Niemenmaa et al., 2008), this method is applicable to many different fungi and serves the purpose intended here. Data on the response‐, residue‐ and error plots, as well as calibration curves and detailed results, are reported in the supplementary materials (Figures S1–S2, Tables S1–S4).

#### 3.1.2 Submerged cultivation of PSS in CPH‐medium

**FIGURE 1:**
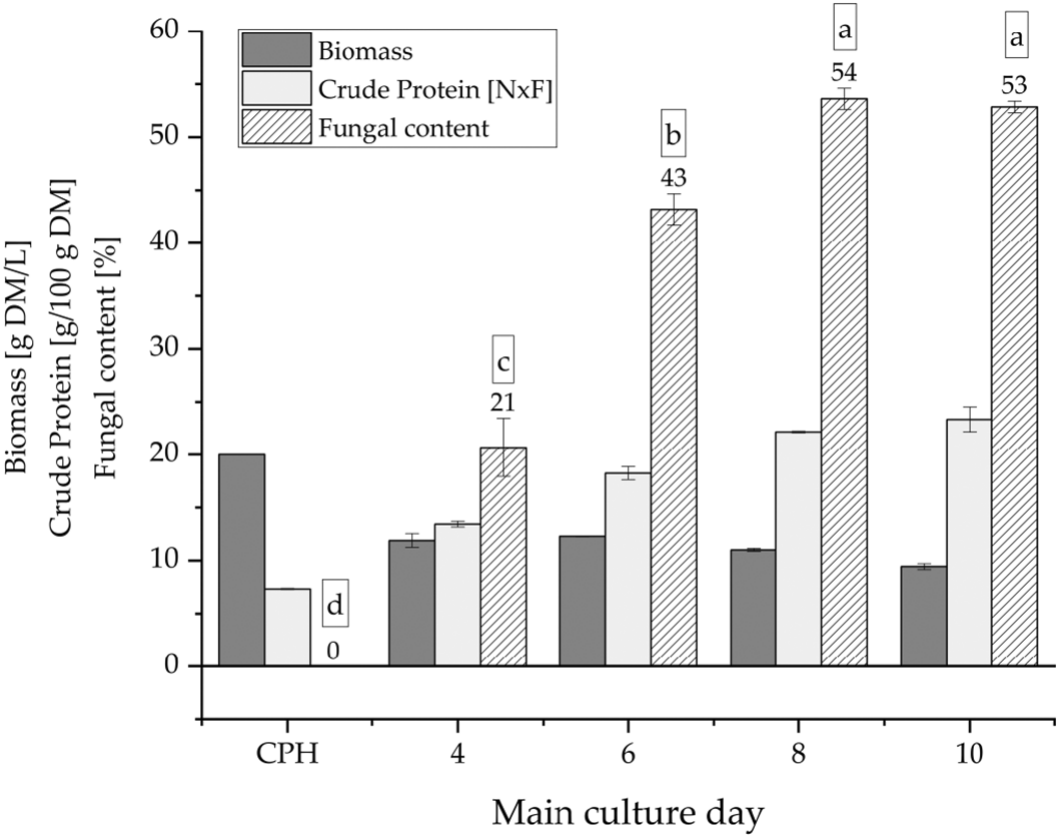
Growth curve of *Pleurotus salmoneo‐stramineus* (PSS) in cocoa pod husk (CPH)‐medium over 10 days (*n* = 2). DM: dry matter; NxF: nitrogen content multiplied by protein factor F. Means with no letter in common indicate significant differences (*p* < .05).

The fermentation experiments were started with 20 g of CPH DM/L. The CPH had a crude protein content of 7.3 ± 0.1 g/100 g DM, and the initial fungal content was 0% since no ergosterol was detected in the CPH. Since ergosterol is found almost exclusively in the cell membranes of fungi, it may serve as a biomarker for fungal growth. Therefore, ergosterol can also be used to exclude unintended fungal infestation of the substrate prior to use as a fermentation substrate (Ibrahim et al., 2022; Osswald et al., 1986). The crude protein content increased until main culture day 8 (up to 22.2 ± 0.1 g/100 g DM; no significant difference to main culture day 10, *p* < .05) as well as the fungal content (up to 54%; 233 mg/100 g DM ergosterol; no significant difference to main culture day 10, *p* < .05) (Figure 1). For economic reasons, a longer cultivation time with consistent results is not beneficial. The dry biomass of the fermentates was approximately halved during fermentation and harvesting. This loss of biomass can be attributed to the dissolution of components during fermentation, which are separated by the harvesting step. The harvest of the non‐inoculated CPH‐medium as a blank yielded a dry biomass of 11.9 ± 0.1 g DM/L. Vriesmann et al. (2011) reported a yield of water‐soluble pectins after aqueous extraction at 100°C of 12.6%. During media preparation, the CPHs were autoclaved for 20 min at 120°C. This represents an aqueous extraction under the influence of high temperatures. Furthermore, CPHs contain between 8.4% and 10.4% reducing sugars and between 9.6% and 11.4% soluble dietary fiber (Vriesmann et al., 2011; Yapo et al., 2013). In addition to the leaching of soluble components, losses of biomass may also be attributed to the fungal metabolism (Kirk et al., 1975). This explains the decrease in recovery of solid biomass with increasing fungal content.

Due to the low nitrogen content of the CPH, it was necessary to supplement the CPH‐based medium with a nitrogen source to improve fungal growth. Fraatz et al. (2014) showed that supplementation with asparagine and aspartic acid may boost the growth of *Pleurotus* species in submerged cultures. Therefore, the optimum amount was determined in preliminary tests by quantifying residual amounts of aspartate in the supernatant at the end of the culture period (data not shown).

The upcycling of different side streams and agricultural wastes with mushrooms has been described in several studies. Ahlborn et al. (2019) upcycled apple pomace with different basidiomycetes and obtained mycelial biomasses with crude protein contents between 9.6% with *Wolfiporia cocos* and 25.4% DM with *Pleurotus sapidus*. The corresponding yields of dry biomass ranged from 13.2–14.5 g DM/L. In a similar study, *Ganoderma lucidum*, *Lentinula edodes*, and *Pleurotus ostreatus* were cultivated on winery waste. Crude protein contents between 17.6 and 19.7 g/100 g DM were achieved (Petre et al., 2016). The results obtained in this study, especially for crude protein content and yield of biomass, are comparable with those from those two studies. In terms of fiber content, CPH lies within common ranges for fruit pomaces. Vriesmann et al. [10] showed a lignin content of 21.4 g/100 g DM in CPH, while apple pomace has a lignin content of 15.4 g/100 g and grape pomace of 56.7 g/100 g DM (Okoro et al., 2021). A study by Manu‐Tawiah and Martin (1987) showed better growth of *Pleurotus ostreatus* on a complex medium (peat‐extract medium) than on a synthetic medium. Recently, basidiomycetes were used to upcycle side streams of the palm oil industry to serve as a rearing substrate for insects. The fermentation positively influenced the development of the insect larvae (Klüber et al., 2022). All mentioned studies, including the present one, demonstrate that basidiomycetes are able to grow on different side stream‐based media. In addition, the protein content of CPHF resembled that of PSS grown on a standard nutrition medium (malt extract) with 26.1 ± 0.1 g/100 g DM. The use of agricultural side streams, such as CPH, is advantageous regarding valorization and cost savings (Bosse et al., 2013). To make this approach even more efficient, replacing the supplementation with aspartic acid with another side stream rich in aspartic acid might be addressed in future studies.

**FIGURE 2:**
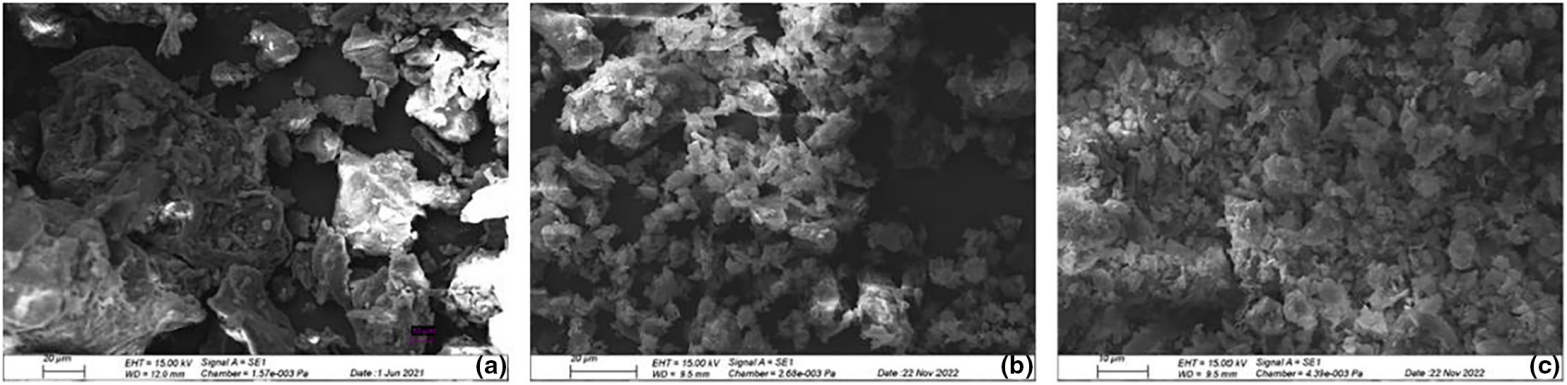
Environmental scanning electron microscopy recordings from cocoa pod husks (CPH) (a), pure *Pleurotus salmoneo‐straimenus* (PSS) mycelium (b), and fermented cocoa pod husks (CPHF) (c).

The surface structures of CPHs were compared to those of CPHF and pure PSS mycelium by means of scanning electron microscopy. The surface structure of CPHs (Figure 2a) differed significantly from the structure after fermentation (Figure 2b), on which fungal mycelium could be observed evenly distributed on the particles' surface. The pure PSS mycelium (Figure 2c) showed composites formed by smaller particles, while the CPH particles were larger and displayed a more uniform surface. Zhu et al. (2016) applied scanning electron microscopy to investigate the morphological changes in Jerusalem artichoke stalks after inoculation with different fungal strains. The different fungal species caused varying levels of degradation to the stalks' cell walls, with *P. chrysosporium* causing the most extensive decay and *G. trabeum* leading to disrupted and weakened cell wall structures. The physical invasion of cell walls by the mycelia through the fissures between cells was assumed to be dependent on degradative mechanisms for polysaccharide depolymerization by the different species. In that regard, the surface coverage of CPH by PSS may suggest a fungal capacity to degrade the husk's woody cell wall matrix, enabling the growth of mycelium in the CPH's porous structures and in intercellular spaces. Moreover, the similarity of CPHF compared to pure PSS mycelium may allow for the detection of fungal growth at the microscopic level.

#### 3.1.3 Evaluation of CPH and CPHF for the development of food products

The DM content of CPH and CPHF accounted for 89.2% and 94.3% (Table 1), respectively, while the DM content of the flour was 86.3%, well in accordance with values reported in the literature (86.7%) (Chandra et al., 2015). The chemical composition of the flour is described in Table S5 in the supplementary materials.

**TABLE 1:** Chemical composition and techno‐functional properties of cocoa pod husks (CPH) and cocoa pod husks fermented with *Pleurotus salmoneo‐stramineus* (CPHF) (*n* = 3).

	CPH	CPHF
Dry matter (DM) [%]	89.2 ± 0.8^a^	94.3 ± 3.8^a^
Crude protein [g/100 g DM]	07.3 ± 0.1^b^	18.9 ± 0.1^a^
Ash [g/100 g DM]	09.1 ± 0.2^a^	04.8 ± 0.1^b^
Protein solubility [%]	14.1 ± 1.8^a^	13.0 ± 0.7^a^
Water‐binding capacity (WBC) [mL/g]	06.2 ± 1.6^a^	03.5 ± 0.1^b^
Oil‐binding capacity (OBC) [mL/g]	1.1 ± 0.1^b^	02.1 ± 0.2^a^
Total dietary fiber [g/100 g DM]	63.6 ± 6.6^a^	73.4 ± 5.4^a^
Insoluble dietary fiber [g/100 g DM]	53.6 ± 4.2^b^	71.1 ± 5.3^a^
Soluble dietary fiber [g/100 g DM]	10.1 ± 0.1^a^	02.3 ± 1.4^b^

*Note*: Means in the same row with no letter in common indicate significant differences (*p* < .05).

Proteins have an extensive influence on the functional, nutritional, and textural properties of raw materials, processed foods, and formulated foods (Zayas, 1997). In general, basidiomycetes exhibit a high protein content and a good protein quality, whether as a fruiting body or mycelium (Berger et al., 2022; Yu et al., 2017). The crude protein content of wheat flour was 9.8 g/100 g DM (Table S5), as reported previously (Belitz et al., 2004). The protein content of CPH was 7.3 g/100 g DM, and it increased by fermentation to 18.9 g/100 g DM in CPHF. The growth of fungal mycelium thus leads to an increased protein content compared to the initial substrate (Ahlborn et al., 2019; Petre et al., 2016), giving the fermentate a significant advantage in terms of nutritional value. The BV of CPH and CPHF was comparably high at 85 and 86, respectively (Table 2). These values are significantly higher than those of soy protein (BV 74) or French beans (BV 58) (Zajul, 2017). The BV of CPHF is comparable with that of pork meat (86) and beef (83) (Rimbach et al., 2015), making it a good alternative for these animal‐based proteins. Zajul (2017) showed BV for basidiomycetes proteins of *Pleurotus sapidus*, grown on isomaltulose‐molassis, of 82; *Pleurotus sajor‐caju*, grown on isomaltulose‐molasses, of 65; and *Lentinula edodes*, grown on carrot pomace, of 66. In this study, we could additionally demonstrate the dependence of protein formation and amino acid composition on the culture medium. Regarding the pure protein content, the value for pure PSS mycelium was slightly higher than for PSS mycelium grown in CPH medium, yet the BV of the pure PSS mycelium accounted only for 75. The share of glutamic acid/glutamine was high in pure PSS mycelium with 31.2%, in contrast to CPH with 14.1% and CPHF with 18.5%. At the same time, the share of essential amino acids was lower. While tryptophane was the first limiting amino acid in CPH and CPHF, the sum of cysteine and methionine was limiting in the pure PSS mycelium, followed by isoleucine and leucine. Fraatz et al. (2014) showed a similar effect for *Pleurotus sapidus*, for which the medium composition significantly influenced the amino acid composition. By calculation of AA_res_, the pure protein content of CPH was quantified to 4.9 ± 0.1 g/100 g DM and to 17.7 ± 0.8 g/100 g DM for CPHF. This underlines that the quantitation of protein, according to Kjeldahl or Dumas, typically overestimates the true protein contents. For fungal mycelia, the chitin content of the cell wall contributes to the total nitrogen content (Di Mario et al., 2008; Tshinyangu & Hennebert, 1996). Regarding the protein solubility, this did not differ significantly between the flour, CPH, and CPHF and ranged between 13.0% and 16.8% (Table S5 and Table 1) and will, hence, not be further discussed. Altogether, despite the lower tryptophane concentration and lower ratio of glutamic acid/ glutamine in CPHF compared to PSS mycelium, the results prove PSS suitable for the fermentation of by‐products of the food industry, resulting in an increase in the total protein concentration and proteins with high BV. PSS fermentations of agricultural side‐streams rich in tryptophane and aspartic acid—also in combination with materials with low concentrations thereof—may offer solutions to reduce supplementation costs and optimize protein yields and protein qualities.

**TABLE 2:** Amino acid composition and biological value of cocoa pod husks (CPH), fermented cocoa pod husks (CPHF), and pure *Pleurotus salmoneo‐stramineus* (PSS) mycelium (*n* = 3).

	Amino acids [g/100 g DM] (% amino acid of total amino acids)			% Amino acid of total amino acids
	CPH	CPHF	PSS mycelium	Reference protein FAO/WHO (1973)
Alanine	0.34 ± 0.01^b^ (5.9%)	1.28 ± 0.01^a^ (6.2%)	1.02 ± 0.01^a^ (4.4%)	‐
Arginine	0.20 ± 0.03^c^ (3.6%)	1.01 ± 0.05^b^ (4.9%)	1.53 ± 0.03^a^ (6.6%)	‐
Aspartic acid/asparagine	0.98 ± 0.02^c^ (17.2%)	2.91 ± 0.15^a^ (14.1%)	1.72 ± 0.05^b^ (7.4%)	‐
Cysteine	0.08 ± 0.00^a^ (1.4%)	0.30 ± 0.01^a^ (1.4%)	0.31 ± 0.00^a^ (1.3%)	‐
Glutamic acid/glutamine	0.80 ± 0.02^c^ (14.1%)	3.82 ± 0.07^b^ (18.5%)	7.21 ± 0.49^a^ (31.2%)	‐
Glycine	0.27 ± 0.00^b^ (4.8%)	0.93 ± 0.04^a^ (4.5%)	0.92 ± 0.02^a^ (4.0%)	‐
Histidine	0.24 ± 0.00^c^ (4.2%)	1.42 ± 0.07^b^ (6.9%)	2.38 ± 0.07^a^ (10.3%)	‐
Isoleucine	0.23 ± 0.00^b^ (4.0%)	0.88 ± 0.05^a^ (4.3%)	0.64 ± 0.03^a^ (2.8%)	4.0%
Leucine	0.39 ± 0.01^c^ (6.8%)	1.51 ± 0.07^a^ (7.3%)	1.12 ± 0.05^b^ (4.8%)	7.0%
Lysine	0.44 ± 0.01^b^ (7.7%)	0.93 ± 0.05^a^ (4.5%)	1.07 ± 0.04^a^ (4.6%)	5.5%
Methionine	0.10 ± 0.00^a^ (1.7%)	0.34 ± 0.01^a^ (1.7%)	0.24 ± 0.01^a^ (1.0%)	‐
Phenylalanine	0.28 ± 0.01^b^ (4.8%)	0.92 ± 0.05^a^ (4.5%)	0.70 ± 0.02^a^ (3.0%)	‐
Proline	0.28 ± 0.01^b^ (4.9%)	0.78 ± 0.04^a^ (3.8%)	0.72 ± 0.02^a^ (3.1%)	‐
Serine	0.34 ± 0.00^b^ (5.9%)	1.07 ± 0.05^a^ (5.2%)	1.02 ± 0.00^a^ (4.4%)	‐
Threonine	0.24 ± 0.00^b^ (4.1%)	0.91 ± 0.05^a^ (4.4%)	1.04 ± 0.01^a^ (4.5%)	4.0%
Tryptophane	0.03 ± 0.01^a^ (0.5%)	0.12 ± 0.01^a^ (0.6%)	0.20 ± 0.00^a^ (0.9%)	1.0%
Tyrsoine	0.17 ± 0.01^a^ (3.0%)	0.34 ± 0.03^a^ (1.7%)	0.39 ± 0.01^a^ (1.7%)	‐
Valine	0.31 ± 0.01^b^ (5.4%)	1.13 ± 0.05^a^ (5.5%)	0.91 ± 0.02^a^ (3.9%)	5.0%
Cysteine+Methionine	0.17 ± 0.00 (3.1%)	0.64 ± 0.01 (3.2%)	0.55 ± 0.02 (2.5%)	3.5%
Phenylalanine+Tyrosine	0.47 ± 0.03 (8.2%)	1.26 ± 0.09 (5.9%)	1.09 ± 0.02 (4.9%)	6%
pure protein (AAres)	4.89 ± 0.10	17.68 ± 0.75	19.99 ± 0.24	‐
BV	85 ± 2	86 ± 0	74 ± 3	100
1st limiting AA	Trp	Trp	Cys + Met	‐
2nd limiting AA	Cys + Met	Lys	Ile	‐
3rd limiting AA	‐	Cys + Met	Leu	‐

*Note*: Means in the same row with different letters indicate significant differences (*p* < .05).

The WBC of a material describes its capacity to retain water under centrifugal pressure or compression. It represents the sum of bound, hydrodynamic, and physically trapped water (López et al., 1996). The water‐binding capacities of flour (Table S5), CPH, and CPHF were determined (Table 1). The WBC of CPH accounted for 6.2 mL/g and was, thus, higher than the WBC of CPHF (3.5 mL/g) and flour (0.62 mL/g). In the literature, values of 0.84 mL/g for flour (Soral‐Śmietana et al., 2003) and of 5.8 mL/g for CPH have been reported (Figueroa et al., 2020). The minor deviations from the values previously reported might be explained by natural variations in the materials. The chemical composition of cocoa, especially the fiber fraction, can be affected by the harvest season, the maturation degree of the fruits, and the cocoa variety (Balladares et al., 2016). Furthermore, the WBC is closely associated with the hydration levels of protein and fiber. Excessive WBC of a particular ingredient can lead to dehydration of other components in formulated foods, negatively affecting the texture, color, and overall sensory properties of the final product. This is the case in baked goods, where the proper hydration of all flour components, especially protein and starch, is essential for the appropriate dough formation (Zayas, 1997). Overall, the WBC of CPH and CPHF were higher than values reported for flours conventionally used in bakery products (Mesías & Morales, 2017), suggesting the use of CPH and CPHF in formulated foods may exert a strong influence on the organoleptic quality and shelf‐life of the final product, such as dry mouthfeel or slower water release rates (syneresis).

The OBC is highly important for the texture and mouthfeel of baked goods, meat formulations, and soups (Belitz et al., 2004). The OBC of flour, CPH, and CPHF accounted for 1.0 mL/g, 1.1 mL/g, and 2.1 mL/g, respectively (Table 1 and Table S5). The OBC capacity of CPH was in accordance with values reported by Figueroa et al. (2020), corresponding to 1.2 mL/g. The OBCs of CPH and wheat flour did not differ statistically from each other. Conversely, CPHF exhibited the highest OBC and differed significantly from CPH. The reason for this might be the larger particle surface areas of CPHF, caused by overall smaller particle size classes compared to CPH (Table 3) (Benítez et al., 2017). The use of CPHF may offer interesting perspectives for the use in foods with large contents of oils, such as chocolate fillings, as they may help reduce unwanted migration of lipids, deaccelerate the formation of fat blooms, and increase the products' shelf‐life (Eibl & Rothkopf, 2018).

**TABLE 3:** Particle size classes (*n* = 6) and L*a*b* (*n* = 3) color components of wheat flour, cocoa pod husks (CPH), and cocoa pod husks fermented with *Pleurotus salmoeo‐stramineus* (CPHF).

	Wheat flour	CPH	CPHF
d_v,0.1_ [μm]	15.27 ± 0.05^b^	19.78 ± 0.80^a^	12.60 ± 0.17^c^
d_v,0.5_ [μm]	67.23 ± 0.34^c^	210.0 ± 16.83^a^	123.33 ± 3.20^b^
d_v,0.9_ [μm]	143.67 ± 1.37^c^	525.67 ± 29.74^a^	373.33 ± 8.11^b^
L* [−]	91.46 ± 0.0^a^	48.82 ± 0.14^b^	40.36 ± 0.19^c^
a* [−]	0.64 ± 0.01^c^	12.70 ± 0.27^a^	12.03 ± 032^b^
b* [−]	9.35 ± 0.0^c^	22.17 ± 0.18^a^	20.55 ± 0.27^b^
Browning index [−]	11.04 ± 0.0^c^	78.08 ± 0.11^b^	90.90 ± 0.83^a^
Color [−]			

*Note*: Means in the same row with different letters indicate significant differences (*p* < .05).

Due to its low caloric content and its health‐promoting benefits, dietary fiber is an important nutrient often lacking in the Western diet. Positive correlations between the amount of ingested fiber and a risk reduction for metabolic and heart diseases have been proposed (Ötles & Ozgoz, 2014). Overall, the amount of total dietary fiber (TDF) in the fermented husks did not differ significantly from CPH. The TDF of CPH accounted for 63.6 g/100 g DM and 73.4 g/100 g DM for CPHF (Table 1). The total carbohydrate and TDF contents of CPH vary widely. TDF values between 18% and 59% of the total cocoa pod weight have been previously reported. Additionally, insoluble dietary fiber (IDF) has been described as the predominant fraction, accounting for 48%, while soluble dietary fiber (SDF) accounts for approximately 11% (Figueroa et al., 2020). The insoluble fiber fraction was predominant in CPH and CPHF. The relative amount of IDF increased with fermentation from 53.6 g/100 g DM in CPH to 71.1 g/100 g DM in CPHF, whereas the relative amount of SDF was reduced from 10.1 g/100 g DM to 2.3 g/100 g DM. The relative increase in TDF, especially in the IDF, can be explained by the solubility of the SDF and, thus, the loss through harvesting. The higher proportion of SDF in the unfermented husks may explain their higher WBC, as water‐soluble fibers often present more hydroxyl groups able to interact with water through hydrogen bonds (Chen et al., 2021). The high fiber content of CPHF, together with its increased protein content compared to the unfermented material, may enable its use as a bulking agent to increase the protein and fiber contents of formulated foods.

**FIGURE 3:**
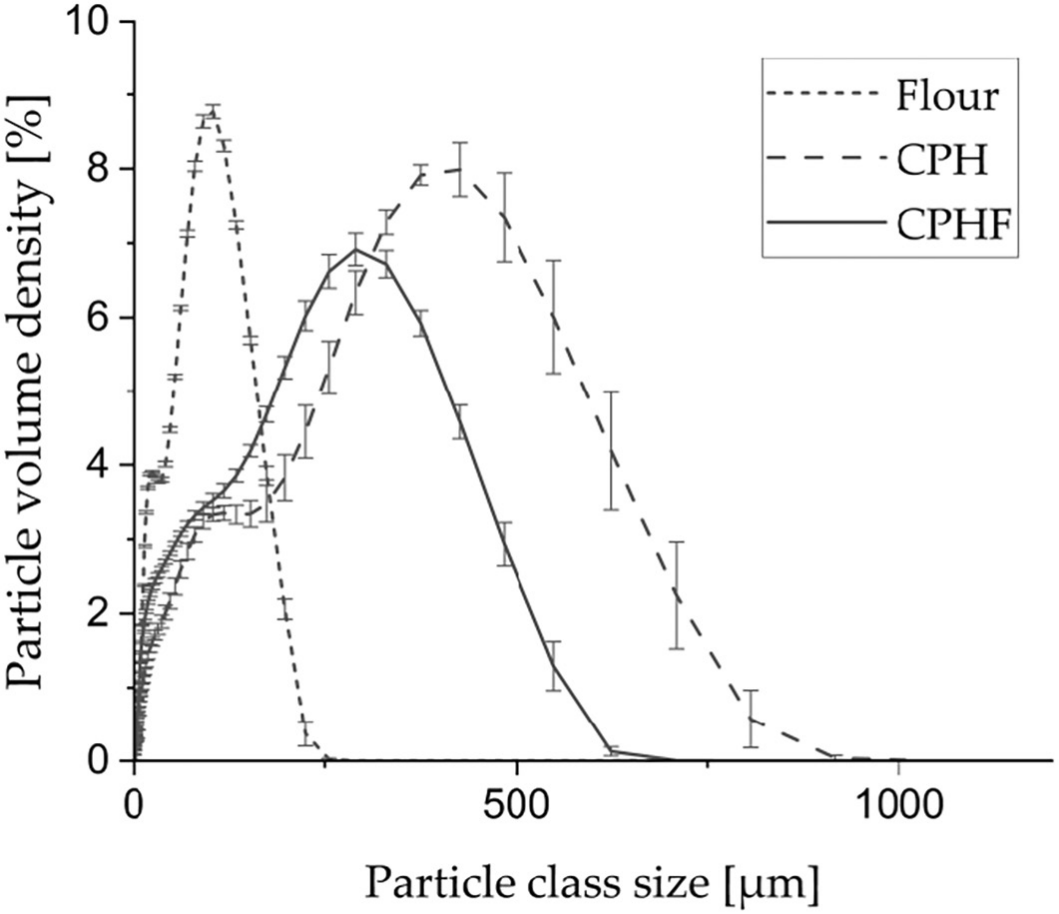
Particle size distribution of white flour, cocoa pod husks (CPH), and fermented cocoa pod husks (CPHF) (*n* = 6).

The particle size distribution indicates the percentile of particles in a certain size class or fraction. The diameters d_v,0.1_, d_v,0.5_, d_v,0.9_ correspond to 10, 50, and 90 vol% on a relative cumulative particle size curve, respectively (Servais et al., 2002). All three samples presented bimodal particle size distribution curves (Figure 3), indicating a higher predominance of two particle size classes on each curve. The d_v,0.9_ of flour was 144 μm, while the d_v,0.9_ of CPH and CPHF were 526 μm and 373 μm, respectively (Table 3). CPH showed a larger d_v,0.9_ compared to CPHF, probably caused by the higher hardness of the material. The d_v,0.9_ of CPHF was likely smaller due to the processing step the pod husks were subjected to. Previous studies have reported on different metabolic mechanisms of fungal species for the degradation of lignin and other cell wall polysaccharides (Zhu et al., 2016). The growth of PSS on the CPH and its putative cell wall degrading activities may have accounted for the disruption of the husks' structures, resulting in finer powders compared to the non‐fermented husks when milled under the same conditions. The microscopic images (Figure 2) demonstrate a complete surface coverage of the fermented husks by mycelium, supporting this assumption.

In addition to the chemical composition, the color of the three samples was measured. The flour's L*a*b* values were 91.46, 0.64, and 9.35, respectively (Table 3). The L* of CPH and CPHF was 48.82 and 40.36, respectively, indicating a darker color of the fermented husks. The a* values of CPH (12.70) and CPHF (12.03) were in a closer range but still statistically different. The b* component of the CPH's color accounted for 22.17 and 20.55 for CPHF. The lower L* value of CPHF was also reflected in a higher browning index, 90.90 compared to 78.08 for CPH. The higher BI of CPHF may be attributed to the growth of the fungal species, an exposure to heat during autoclavation, and hence the formation of oxidation products as well as ongoing enzymatic activities. To understand the color differences better, further analyses of the composition of the CPH and the CPHF are needed and should be the focus of future studies.

### 3.2 Textural properties of four blends and bread doughs with different concentrations of CPH and CPHF

Bread is a staple food that is commonly consumed in many countries. Traditional bread recipes primarily rely on wheat flour, which is produced by removing the bran and germ fractions of the wheat grain. By doing so, important nutrients are lost, and the bread made from white flour is limited in dietary fibers (Xu et al., 2019). Therefore, we investigated the addition of CPH and CPHF to bread doughs as a means to increase the protein and dietary fiber content of white bread.

**FIGURE 4:**
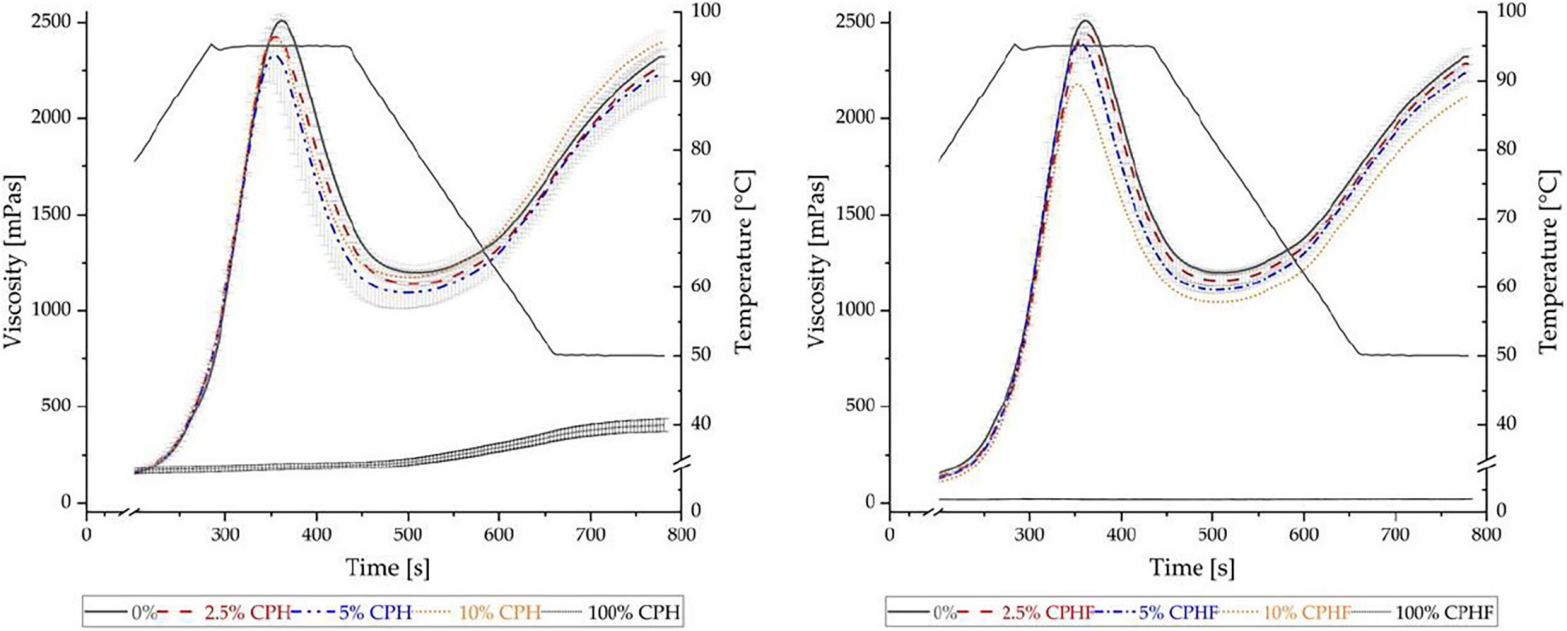
Pasting properties of flour blends with 0%—100% addition of cocoa pod husks (CPH) (left) and fermented cocoa pod husks (CPHF) (right) (*n* = 3).

The pasting properties of flour blends give important insights on the breads' final characteristics (Fu et al., 2008). Overall, the pasting temperature, which describes the temperature at which the viscosity begins to increase during the heating process, stayed constant for all flour‐blends containing CPH as well as CPHF (Figure 4). A high peak viscosity (PV) hints at a high water‐holding capacity (Balet et al., 2019). In spite of the higher WBC of the unfermented husks compared to CPHF, flour samples containing CPH did not exhibit a clear trend, besides showing lower PV compared to the flour. As wheat flour is mixed with water, a three‐dimensional structure is created, where gluten particles are integrated into membranes that contain granules of starch and other flour components (Fu et al., 2008; Zayas, 1997). Therefore, the lower PV of the CPH samples may indicate an impediment in the formation of this network, resulting in a lower degree of starch‐swelling and, thus, lower PV. The unfermented husks alone did not show a conventional pasting curve, hinting at a low starch concentration therein. The light increase in viscosity after thermal input may be explained by the presence of pectin in CPH. Cocoa husks have been reported to contain approximately 6% pectin (Sobamiwa & Longe, 1994). The gelling point of pectin has been determined to range between 51 and 86°C (Kastner et al., 2012).

The PV of samples with CPHF decreased with an increasing level of substitution. In the sample with pure CPHF, the viscosity remained constant throughout the pasting experiment. As the water was in abundance, it is possible that the fibers, predominantly water‐insoluble, were completely hydrated and that their capacity for water uptake was already at its limit, resulting in a constant viscosity (Zhou et al., 2021). Furthermore, the final viscosity of 10% CPH was higher compared to the sample with equal CPHF concentration, possibly due to the higher WBC of the unfermented husks and a stronger competition for the water between the CPH and the flour. Two hypotheses have been proposed for the interaction of fibers with gluten networks. The first suggests that the dietary fiber competitively binds water, leading to the partial dehydration of gluten. Consequently, this induces a conformational change in the gluten matrix and causes the collapse of the polymeric network of gluten. According to this, the detrimental effects of fibers in dough can be mitigated by optimizing the water content. The second hypothesis hints at a gluten‐diluting effect, whereby fibers physically disrupt the gluten network. This disruption occurs when fibers interfere with the cohesive structure of gluten, leading to its dispersion or the dispersion of gluten aggregates (Zhou et al., 2021). Therefore, as CPH and CPHF contained considerable amounts of dietary fiber, reduced gluten network formation due to the competition for water and physical hindrance by the fibers are possible.

Texture profile analyses of bread doughs containing 0% (*w/w*), 2.5% (*w/w*), 5% (*w/w*), and 10% (*w/w*) CPH and CPHF were performed. A replacement with 2.5% (2.24 N) and 5% CPH (2.25 N), as well as with 2.5% CPHF (1.54 N), led to a reduced dough hardness compared to the standard bread dough composed of only flour and water (4.25 N). The addition of 10% CPH (3.62 N) as well as 5% CPHF (4.64 N) led to a dough hardness similar to that of standard bread dough. The addition of 10% CPHF increased the hardness to 7.14 N. The samples' springiness was similarly high in all doughs with CPH and in the one with 2.5% CPHF, whereas the samples with 5% CPHF and 10% CPHF were similar to the standard dough. The hardest sample (10 % CPHF) presented the lowest springiness (25.66%). This was followed by 5% CPHF with 31.41% springiness and the white bread dough with 38.72% springiness. The three samples did not differ significantly from each other. However, they were distinct from the samples with 2.5% CPH (89.77%), 5% CPH (93.90%), 10% CPH (88.36%), and 2.5% CPHF (95.52%). The addition of different concentrations of CPH led to elasticities above those of the white bread doughs. As aforementioned, it is likely that the higher WBC of the CPH caused the water to be strongly bound to the CPH's fibers, limiting the unfolding of the gluten proteins and decreasing the degree of cross‐linking between cysteine groups (Gras et al., 2001). Nawrocka et al. (2020) reported on the effect of insoluble and soluble fibers on disulfide‐bond formation. The incorporation of more IDF into doughs led to a more intense abnormal folding of gluten protein. Fiber samples with high SDF content increased the number of stable cysteine bonds (SS bonds), whereas dough weakening, an indication of SS rupture, could be assigned to a high IDF content. As CPHF contained a higher IDF to SDF ratio compared to CPH, the increasing dough hardness and reduced springiness with increasing concentration may be attributed to a higher degree of destabilization of the gluten network by interference in the formation of stable cysteine bonds. Nonetheless, it is important to note that the kneading time and the amount of added water were kept constant, possibly causing the CPH doughs to remain partially undeveloped (Mirsaeedghazi et al., 2008). Experiments producing CPH and CPHF doughs with varying kneading times and water concentrations, as well as studies of the dough's rheological properties, may help elucidate the interactions within the doughs. These should be the focus of future studies.

In a preliminary trial, the optimal water concentration for the wheat flour type 550 was determined. The water necessary to develop the dough accounted for 53.5% (*w/w*) and was in accordance with values previously reported (54.1%) (Nikolic et al., 2013). All doughs developed consistencies above the 500 FE standard range (Table 4). Overall, the maximum resistance, expressed in FE, increased with increasing flour substitution. The addition of CPH led to higher torque values (higher consistency values) compared to CPHF. While 2.5% CPH showed a consistency of 818.5 FE, 2.5% CPHF addition led to a consistency of 808 FE. The differences between CPH‐ and CPHF‐containing doughs became clearer with increasing concentration. Doughs made with 5% CPH showed 942.5 FE, and doughs made with 10% CPH showed 1080.5 FE, whereas doughs produced with 5% CPHF and with 10% CPHF showed values of 871 FE and 945.5 FE, respectively. The dough development time (DT) was also affected by the addition of CPH and CPHF. The DT was slightly shortened in the dough produced with 2.5% CPH, whereas it was prolonged for the other concentrations of either CPH or CPHF compared to white bread dough. This parameter describes the time it takes for the developing dough to reach maximum consistency. Therefore, the longer development times of the substituted doughs suggest it takes more kneading time to integrate the CPH and CPHF into the doughs. The larger particle size classes of CPH and CPHF compared with the flour may have played a role in prolonging the DT. In a study on bread doughs enriched with grape pomace fibers of different particle sizes, Mironeasa et al. (2019) described the effect of particle size on water absorption (WA). The authors observed a longer DT in doughs with larger particle size types. This was attributed to slower water absorption by the larger particles, hampering the formation of the gluten network and increasing the time required for optimal dough development. Further studies using CPH and CPHF powders with finer particle sizes should be carried out in the future. In addition, the equipment's software proposed values for the water absorption to obtain doughs with 500 FE consistency. These were 61.4%, 64.6%, and 67.9% for 2.5%, 5%, and 10% CPH, respectively. The water absorption values for CPHF were 62.8%, 63.1%, and 64.7% for 2.5%, 5%, and 10% addition, respectively. A contributing factor for the higher water amounts recommended might be the higher protein content of the studied flour blends compared to the wheat flour. Thus, by replacing a part of the flour with CPH or CPHF, the relative amount of protein in the dough increased (Nikolic et al., 2013). Another factor to consider are changes in the fiber fraction of the doughs as well as their concentrations therein. Dietary fiber may increase water absorption, mainly due to the higher number of hydroxyl groups allowing for a stronger interaction with water through hydrogen bonding (Kurek et al., 2016). Consequently, there is stronger competition between the added proteins, fibers, and gluten in the available water. Future trials carried out with adapted water concentrations are necessary to determine if consistency more similar to an ideal standard for bread dough can be achieved.

**TABLE 4:** Farinographic properties of bread doughs made with 0% (*w/w*), 2.5% (*w/w*), 5% (*w/w*), and 10% (*w/w*) cocoa pod husks (CPH) and fermented cocoa pod husks (CPHF) (*n* = 2).

Description	Flour	CPH 2.5%	CPH 5%	CPH 10%	CPHF 2.5%	CPHF 5%	CPHF 10%
Water absorption (WA) [%]	53.3 ± 0.3	53.4 ± 0.1	53.5 ± 0.1	53.35 ± 0.1	53.5 ± 0.1	53.8 ± 0.1	53.6 ± 0.2
Development time [mm:ss]	00:01:46 ± 0:00:06	00:01:40 ± 0:00:23	00:03:50 ± 0:01:26	00:05:39 ± 0:02:10	00:03:26 ± 0:02:41	00:06:01 ± 0:03:00	00:11:57 ± 0:02:41
Consistency [FE]	511 ± 6.4	818.5 ± 82.7	942.5 ± 132.2	1080.5 ± 181.7	808 ± 138.6	871 ± 151.32	945.5 ± 115.3
WA consistency [%]	54.1 ± 1.1	61.4 ± 1.9	64.6 ± 3.5	67.9 ± 4.5	62.8 ± 5.8	63.1 ± 3.9	64.7 ± 2.7
Dough stability [mm:ss]	0:01:17 ± 0:00:43	00:02:46 ± 0:01:02	00:04:54 ± 0:00:28	00:03:27 ± 0:00:57	00:04:41 ± 0:04:20	0:06:39 ± 0:02:24	00:07:13 ± 0:00:20

The addition of CPH and CPHF caused the bread dough to change color (Table S6). With increasing concentrations of CPH and CPHF, the L* value of the dough decreased, indicating the dough became darker. CPHF had a stronger effect on this parameter compared to CPH. The same trend was observed for the a* component. On the other hand, the b* component was higher in dough made with 10% CPH. The b* values of doughs containing 10%, 5%, and 2.5% CPHF, and 5% CPH were commensurate. The changes in the L*a*b* values were also reflected in the BI of the doughs. Browning became higher with increasing concentrations of CPH and CPHF. CPHF caused the samples to darken more than CPH. The dark color of the dough may present a challenge in the use of CPHF as a food ingredient. There are, however, positive associations between brown food products, especially baked goods, and their acceptance by consumers, as these are sometimes associated with a higher fiber content and possible health‐promoting benefits (Clydesdale, 1993; Downey, 2011). The belief that a darker color suggests a healthier product has encouraged the bakery industry to increase the use of food colorants (Downey, 2011). Therefore, CPHF could also be used as a natural food colorant (Grob et al., 2021).

**FIGURE 5:**
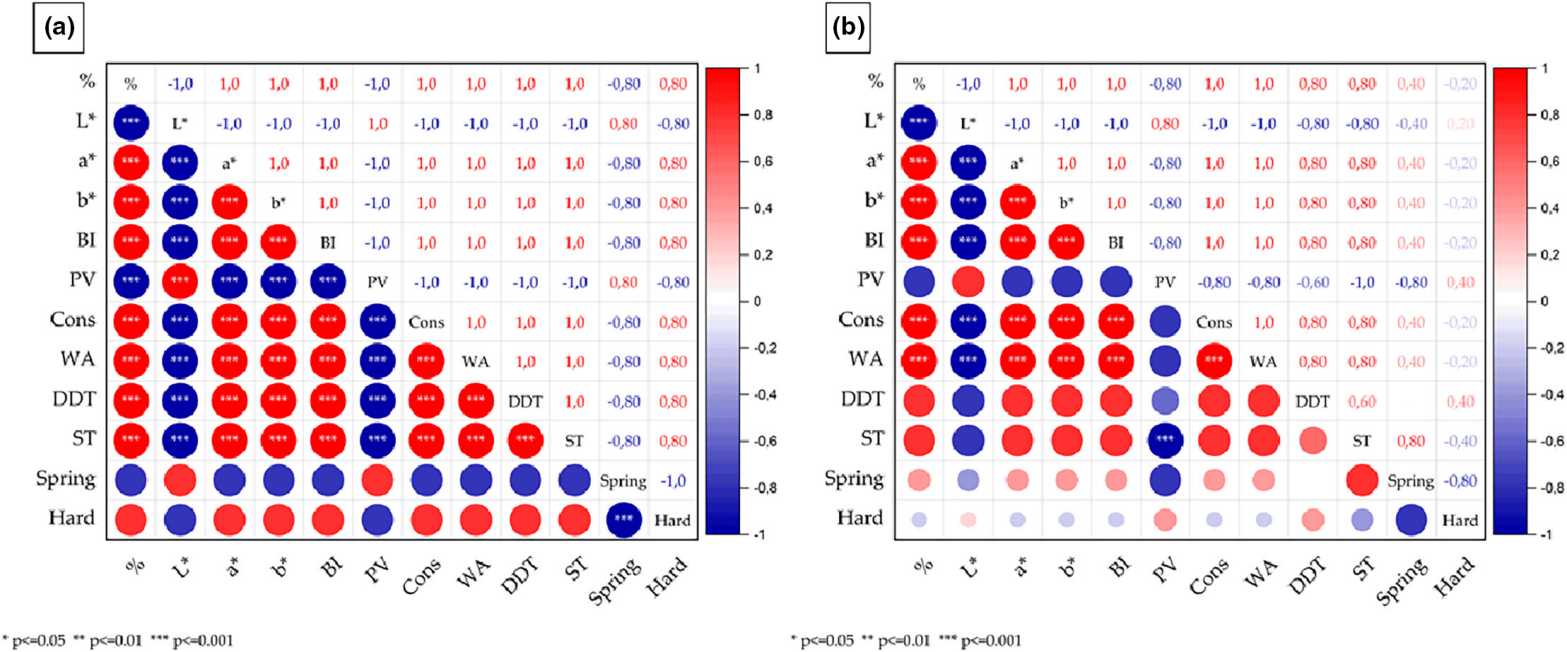
Spearman correlation matrices of dough properties produced with different concentrations of (a) cocoa pod husks (CPH) and (b) fermented cocoa pod husks (CPHF). % indicates the flour substitution in % (*w/w*), BI the browning index, PV the peak viscosity, Cons the consistency, WA the water absorption, DDT the dough determination time, ST the stability time, Spring the springiness, and Hard the hardness of the developed doughs. Red indicates a positive and blue indicates a negative association between the variables.

In accordance with the results previously discussed, Spearman correlation analyses showed a positive association between the concentration of added CPH and the BI, the consistency, the water absorption, the dough development time, and the stability time (Figure 5a). Negative associations were determined between the concentration of CPH and the L* value and between the concentration and the PV. The darkening of wheat bread with the increasing addition of cocoa pod husk powders (CPHPs) was previously reported (Zamri et al., 2013). The PV and the water absorption, as well as the consistency and the PV, were also negatively associated, possibly relating to the reduction of starch and gluten—the main responsible components for the formation of networks in wheat doughs—by dietary fibers. Moreover, the correlation analyses indicated a negative association between the hardness and the springiness. In addition, an increase in dough development time and water absorption can often be observed in dough formulations enriched with fibers. The strength and stickiness of such doughs are expected to be increased, while the tolerance for mixing and fermentation is diminished (Zhou et al., 2021). This was described for breads produced with CPHPs. The authors reported: “as the concentration of CPHP increased in the formulation, the specific volume, microscopic structure, and pore size of bread underwent a decrease, leading to an observable rise in the bread's final hardness” (Zamri et al., 2013). Furthermore, Spearman correlations suggested a significant positive relationship between the concentration of CPHF and the a*‐value, the b*‐value, the BI, the consistency, and the water absorption (Figure 5b). Similar to CPH, the concentration was negatively related to the L* value. The browning index was shown to be positively linked to the consistency and the water absorption. This was expected, as the three variables are dependent on the concentration of CPHF. Alike observations were made by Ulziijargal et al. (2013), where the replacement of 5% of wheat flour with mushroom mycelium powder led to bread loaves with lower lightness and white index values. Contrarily to CPH, the hardness and springiness were not significantly correlated in samples containing CPHF, possibly due to differences in the fiber compositions between the two ingredients.

## 4 CONCLUSIONS

This paper explored the valorization of cocoa pod husks by fermentation with *Pleurotus salmoneo‐stramineus* as an innovative approach to creating protein‐rich and fiber‐rich ingredients for the food industry. The fermentation conditions were successfully optimized, and the techno‐functional properties of the fermented cocoa pod husks (CPHF) were evaluated for use in food products. We demonstrated that basidiomycetes are able to grow on cocoa pod husks and produce high‐quality protein. We additionally proved the dependence of protein formation and amino acid composition on the culture medium, showing that for PSS, the CPH medium led to an improved protein quality compared to malt extract medium while maintaining the same protein content. The integration of different concentrations of CPH and CPHF in bread doughs was possible, yet their suitability for industrially produced bakery products still needs to be further explored. Experiments producing doughs containing CPH and CPHF by varying the kneading times and the water concentrations added may help elucidate the interactions within the developed doughs and improve the doughs' consistencies. By adding value to cocoa pod husks through fungal fermentation, cocoa farmers may profit from alternative sources of income and improve their livelihoods, fomenting additionally a more sustainable cocoa production.

## AUTHOR CONTRIBUTIONS

**Thomas Bickel Haase:** Conceptualization (equal); formal analysis (equal); investigation (equal); methodology (equal); writing – original draft (equal); writing – review and editing (equal). **Victoria Klis:** Conceptualization (equal); formal analysis (equal); investigation (equal); methodology (equal); writing – original draft (equal). **Andreas Klaus Hammer:** Methodology (equal); validation (equal). **Claudia Pinto Lopez:** Formal analysis (equal); investigation (equal); validation (equal). **Christoph Verheyen:** Writing – review and editing (equal). **Susanne Naumann‐Gola:** Funding acquisition (equal); project administration (equal); supervision (equal); writing – review and editing (equal). **Holger Zorn:** Funding acquisition (equal); project administration (equal); resources (equal); supervision (equal); validation (equal); writing – review and editing (equal).

## FUNDING INFORMATION

The project on which this paper is founded was funded by the German Federal Ministry of Education and Research (BMBF) under grant number 031B0819. The responsibility for the content of this publication lies with the authors.

## CONFLICT OF INTEREST STATEMENT

The authors declare no conflict of interest.

## Supporting information


Appendix S1.

## Data Availability

The data presented in this study are available on request from the corresponding author.
