# Supplementary material for: Fermentation of cocoa pod husks with Pleurotus salmoneo‐stramineus for food applications
Source: Food Sci Nutr. 2024 Jan 12;12(4):2551–66. doi: 10.1002/fsn3.3937 (PMC11016415; doi:10.1002/fsn3.3937)
Supplement: Supplementary file 1 — Appendix S1. [file FSN3-12-2551-s001.docx]

**Supplementary materials**

Fermentation of cocoa pod husks with *Pleurotus salmoneo-stramineus* for food applications

Thomas Bickel Haase ^1, 3, †^, Victoria Klis ^2, 3, †^, Andreas Klaus Hammer ^2^, Claudia Pinto Lopez ^1^, Christoph Verheyen ^1^, Susanne Naumann-Gola ^1^ and Holger Zorn ^2, 3, *^

^1^ Fraunhofer Institute for Process Engineering and Packaging IVV, Giggenhauser Straße 35, 85354 Freising, Germany

^2^ Fraunhofer Institute for Molecular Biology and Applied Ecology IME, Ohlebergsweg 12, 35392 Giessen, Germany

^3^ Institute of Food Chemistry and Food Biotechnology, Justus-Liebig University, Heinrich-Buff-Ring 17, 35392 Giessen, Germany

^*^Correspondence: [holger.zorn@uni-giessen.de](mailto:holger.zorn@uni-giessen.de); Tel.: +49 641 97219130


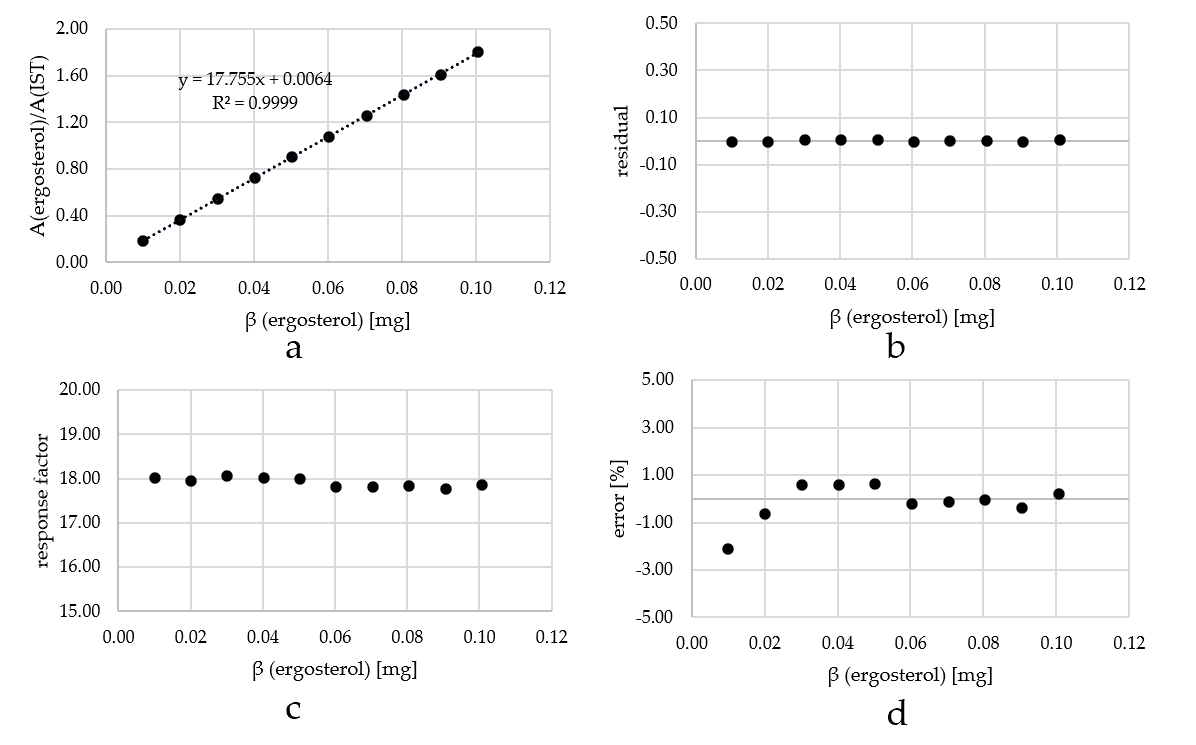


Figure S1a Calibration curve (a), residual plot (b), response factor plot (c) and error plot (d) for basic calibration and linearity proof (a).


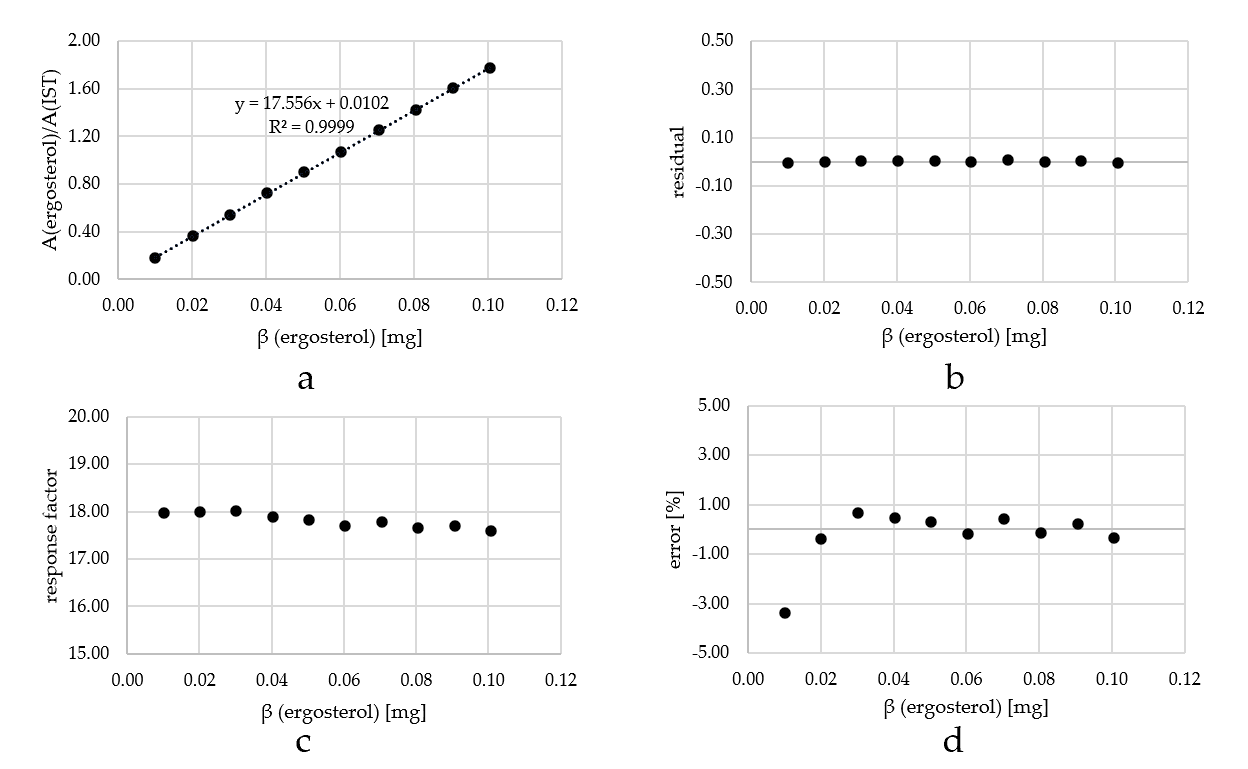


Figure S1b Calibration curve (a), residual plot (b), response factor plot (c) and error plot (d) for basic calibration and linearity proof (b).


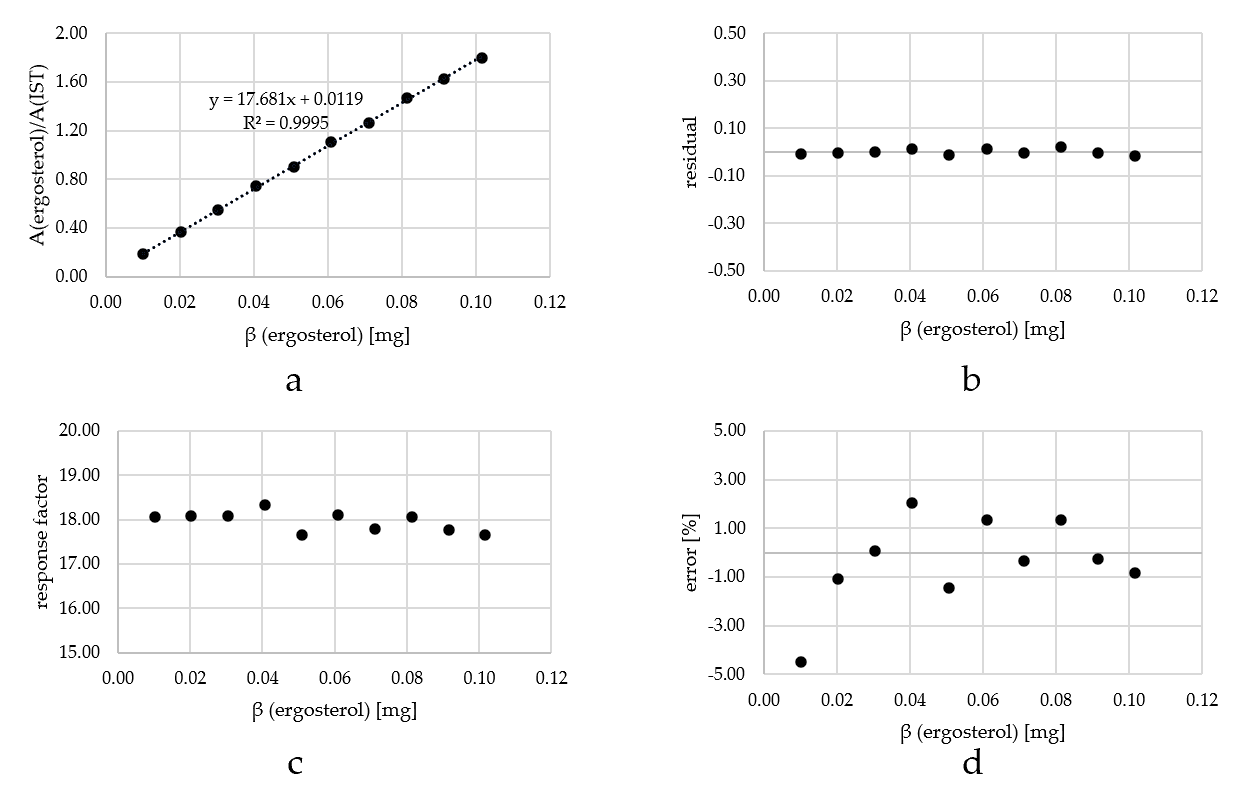


Figure S1c Calibration curve (a), residual plot (b), response factor plot (c) and error plot (d) for basic calibration and linearity proof (c).

Table S1 Mandel’s fitting test/ proof of linearity (F-Test: α=0.01; f_1_=1; f_2_=N-3; N=10).

|  | a | b | c |
| --- | --- | --- | --- |
| test value | 0.62 | -6.63 | 2.43 |
| F | 12.25 | 12.25 | 12.25 |
| conclusion | linear | linear | linear |

Table S2 Recovery in the working range.

|  | a) recovery [%] | b) recovery [%] | c) recovery [%] | $\bar{\text{recovery}}$ [%] |
| --- | --- | --- | --- | --- |
| 10 µg/mL | 92.09 | 98.34 | 101.05 | 97.16 ± 4.59 |
| 20 µg/mL | 96.74 | 98.39 | 101.32 | 98.82 ± 2.32 |
| 30 µg/mL | 105.45 | 100.05 | 99.98 | 101.83 ± 3.14 |
| 40 µg/mL | 99.41 | 100.05 | 99.19 | 99.55 ± 0.45 |
| 50 µg/mL | 99.81 | 100.95 | 100.93 | 100.56 ± 0.66 |
| 60 µg/mL | 100.39 | 100.38 | 99.31 | 100.03 ± 0.62 |
| 70 µg/mL | 100.51 | 99.80 | 99.64 | 99.98 ± 0.47 |
| 80 µg/mL | 100.29 | 100.96 | 99.49 | 100.25 ± 0.73 |
| 90 µg/mL | 100.37 | 99.38 | 99.81 | 99.85 ± 0.50 |
| 100 µg/mL | 98.99 | 99.68 | 100.74 | 99.80 ± 0.88 |


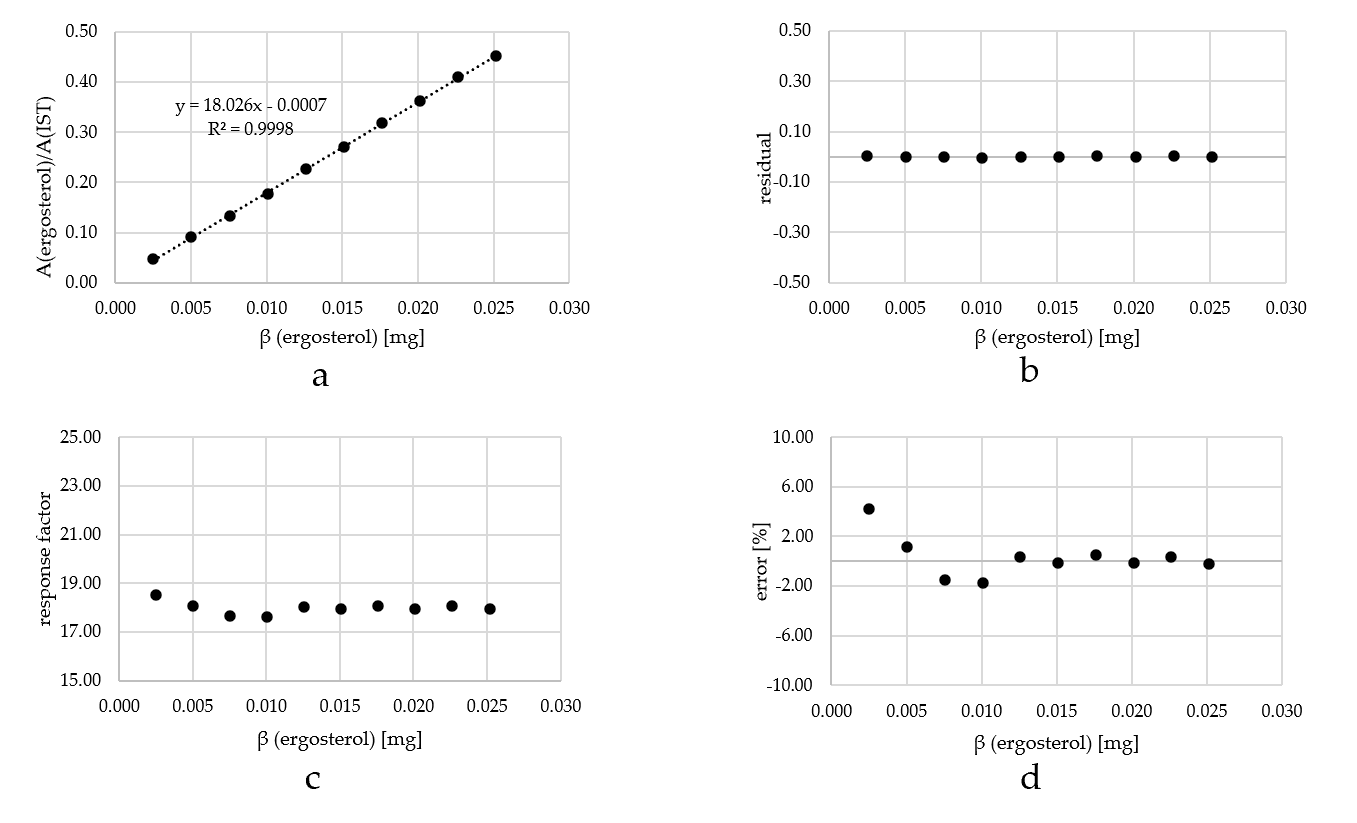


Figure S2a Calibration curve (a), residual plot (b), response factor plot (c) and error plot (d) for LOD and LOQ determination (a).


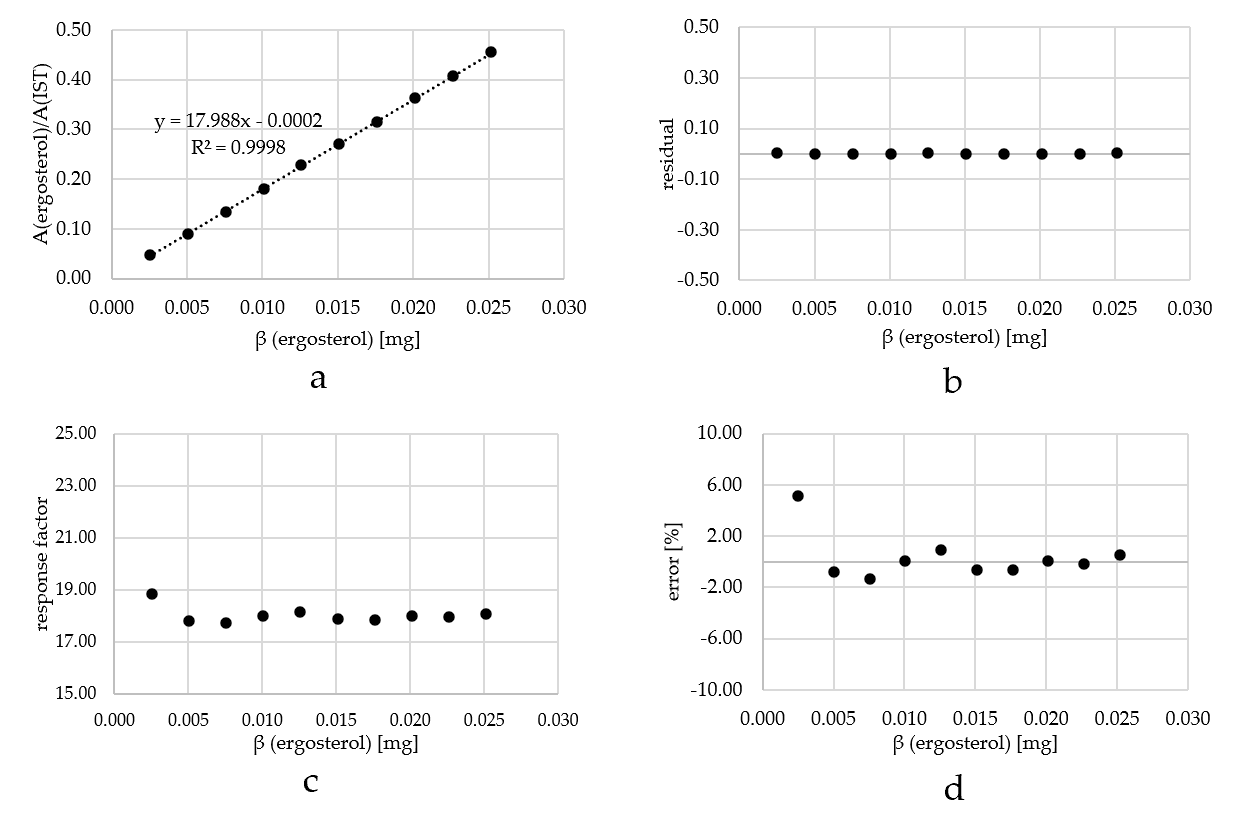


Figure S2b Calibration curve (a), residual plot (b), response factor plot (c) and error plot (d) for LOD and LOQ determination (b).


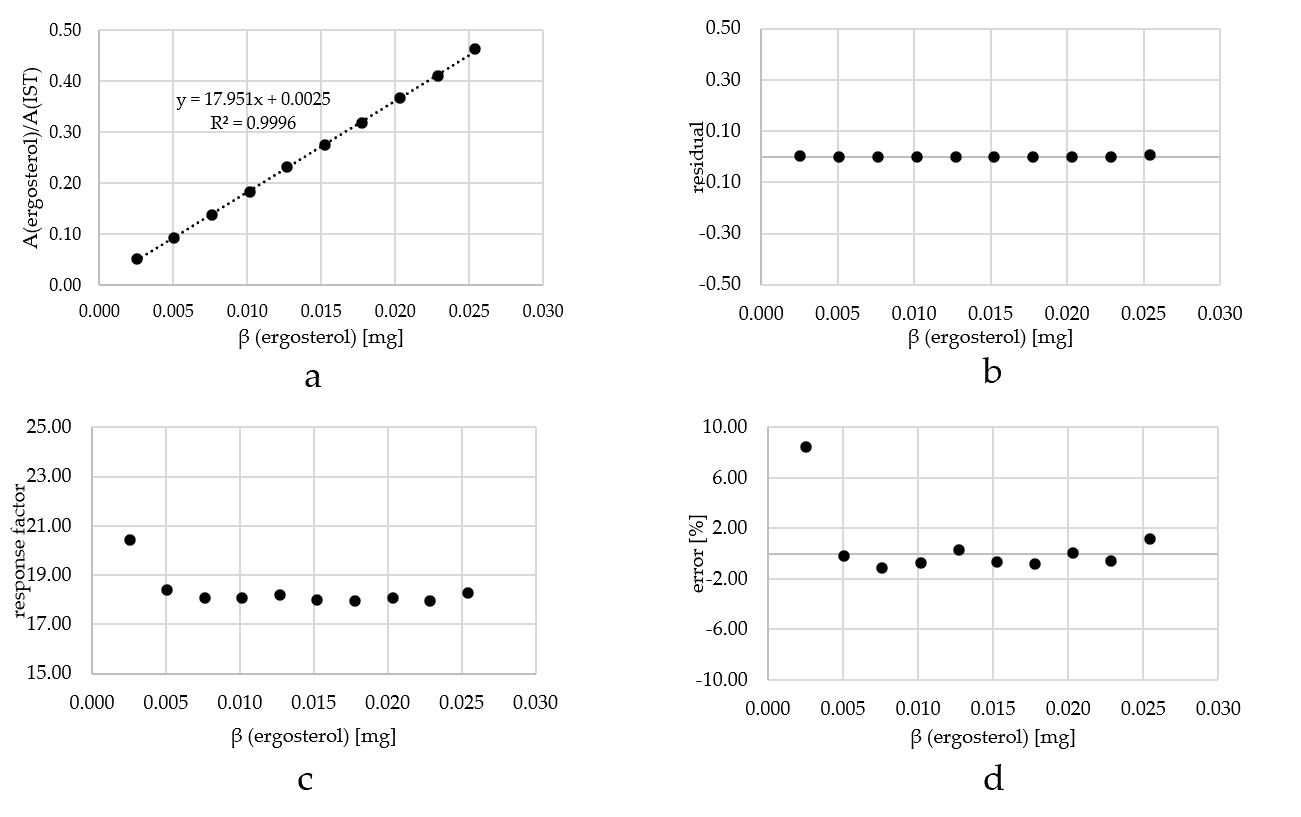


Figure S2c Calibration curve (a), residual plot (b), response factor plot (c) and error plot (d) for LOD and LOQ determination (c).

Table S3 Method precision, 6-fold sample measurement of fermented cocoa pod husks (CPHF).

|  | ergosterol  [mg/ 100 g DM] | average  [mg/ 100 g DM] | standard deviation  [mg/ 100 g DM] | RSD [%] |
| --- | --- | --- | --- | --- |
| a | 252.6 | 252.6 | 10.0 | 4.0 |
| b | 239.8 |  |  |  |
| c | 253.8 |  |  |  |
| d | 244.0 |  |  |  |
| e | 268.1 |  |  |  |
| f | 257.6 |  |  |  |

Table S4 Limit of detection and limit of quantification, results according to DIN 32645 (n=3).

|  | a | b | c | average | [mg/100 g] § |
| --- | --- | --- | --- | --- | --- |
| LOD [µg/mL] | 0.35 | 0.37 | 0.55 | 0.42 ± 0.11 | 2.12 ± 0.57 |
| LOQ [µg/mL] | 1.20 | 1.24 | 1.88 | 1.44 ± 0.38 | 7.22 ± 1.88 |

§ related to 50 mg of sample

Table S5 Chemical composition and technofunctional properties of wheat flour

|  | Wheat flour |
| --- | --- |
| Dry matter (DM) [%] | 86.3 ± 2.4 |
| Crude protein [g/100 g DM] | 9.8 ± 0.1 |
| Ash [g/100 g DM] | 0.9 ± 0.0 |
| Protein solubility [%] | 16.8 ± 1.4 |
| Water-binding capacity (WBC) [mL/g] | 0.6 ± 0.0 |
| Oil-binding capacity (OBC) [mL/g] | 1.0 ± 1.1 |
| Total dietary fibre [g/100 g DM] | 2.4 ± 1.2 |
| Insoluble dietary fibre [g/100 g DM] | 0.9 ± 0.60 |
| Soluble dietary fibre [g/100 g DM] | 1.4 ± 0.6 |

Table S6 Colour of bread doughs produced with 0% (w/w), 2.5% (w/w), 5% (w/w) and 10% (w/w) cocoa pod husks (CPH) and fermented cocoa pod husks (CPHF) expressed as L*a*b values and browning indexes (n=3). Means in the same row with no letter in common indicate significant differences (p < 0.05)

|  | Flour | CPH 2.5% | CPH 5% | CPH 10% | CPHF 2.5% | CPHF 5% | CPHF 10% |
| --- | --- | --- | --- | --- | --- | --- | --- |
| L* [-] | 80.34 ± 0.27 ^a^ | 67.80 ± 0.08 ^b^ | 60.43 ± 0.21 ^c^ | 50.16 ± 0.74 ^e^ | 58.19 ± 0.23 ^d^ | 50.62 ± 0.21 ^e^ | 41.97 ± 0.35 ^f^ |
| a* [-] | 3.66 ± 0.40 ^f^ | 4.64 ± 0.40 ^e^ | 5.81 ± 0.33 ^d^ | 9.20 ± 0.02 ^b^ | 7.40 ± 0.04 ^c^ | 9.29 ± 0.03 ^b^ | 10.82 ± 0.03 ^a^ |
| b* [-] | 11.30 ± 0.08 ^d^ | 12.36 ± 0.31 ^c^ | 14.96 ± 0.35 ^b^ | 16.51 ± 0.33 ^a^ | 14.57 ± 0.30 ^b^ | 15.15 ± 0.29 ^b^ | 14.97 ± 0.29 ^b^ |
| BI [-] | 18.19 ± 0.49 | 24.77 ± 0.54 ^f^ | 34.44 ± 1.88 ^e^ | 52.77 ± 0.63 ^c^ | 37.69 ± 0.78 ^d^ | 48.50 ± 0.91 ^c^ | 62.27 ± 0.60 ^a^ |
|  |  |  |  |  |  |  |  |
